# Occurrence and Antioxidant Activity of C1 Degradation Products in Cocoa

**DOI:** 10.3390/foods6030018

**Published:** 2017-02-28

**Authors:** Cédric De Taeye, Marie-Lucie Kankolongo Cibaka, Sonia Collin

**Affiliations:** Unité de Brasserie et des Industries Alimentaires, Earth and Life Institute, ELIM, Faculté des Bioingénieurs, Université catholique de Louvain, Croix du Sud, 2 bte L07.05.07, B-1348 Louvain-la-Neuve, Belgium; cedric.detaeye@uclouvain.be (C.D.T.), marie-lucie.kankolongo@uclouvain.be (M.-L.K.C.)

**Keywords:** Procyanidin C1, epimers, online TEAC, model medium, flavan-3-ols

## Abstract

Procyanidin C1 is by far the main flavan-3-ol trimer in cocoa. Like other flavan-3-ols, however, it suffers a lot during heat treatments such as roasting. RP-HPLC-HRMS/MS(ESI(−))analysis applied to an aqueous model medium containing commercial procyanidin C1 proved that epimerization is the main reaction involved in its degradation (accounting for 62% of degradation products). In addition to depolymerization, cocoa procyanidin C1 also proved sensitive to oxidation, yielding once- and twice-oxidized dimers. No chemical oligomer involving the native trimer was found in either model medium or cocoa, while two C1 isomers were retrieved. C1 degradation products exhibited antioxidant activity (monitored by RP-HPLC-Online TEAC) close to that of C1 (when expressed in µM TE/mg·kg^−1^).

## 1. Introduction

Procyanidins, also called condensed tannins, are a group of phytoallexins widely distributed within the plant kingdom and composed mainly of flavan-3-ol monomers and oligomers. These chemicals have several health benefits, linked notably to antioxidant, antibacterial, anticancer, and antidiabetic effects [1,2,3,4,5,6,7]. Procyanidins are well represented in cocoa, making it one of the richest sources in the human diet [8].

Procyanidin C1 ((−)-epicatechin-(C4-C8)-(−)-epicatechin-(C4-C8)-(−)-epicatechin) is the main flavan-3-ol trimer found in cocoa. It is also found in cinnamon, tea, apple, litchi, grape, peach, and various red and blue berries [9]. The composition of the procyanidin pool varies according to the availability of flavan-3-ol monomers. Procyanidin C2 ((+)-catechin-(C4-C8)-(+)-catechin-(C4-C8)-(+)-catechin) is another B-type trimer, absent from cocoa but found in high quantity in barley and grapes, where (+)-catechin is the main monomeric building block [10,11].

An additional C2-O-C7 ether bond turns B-type into A-type procyanidins, the second most common type of procyanidins in plants. B2 can thus convert to A2, and conversion of B-type to A-type trimers is also reported in the literature. To date, two once-oxidized trimers, primarily evidenced in cinnamon, are known: cinnamtannin B1 ((−)-epicatechin-(C4-C8,C2-O-C7)-(−)-epicatechin-(C4-C8)-(−)-epicatechin) and cinnamtannin D1 ((−)-epicatechin-(C4-C8,C2-O-C7)-(−)-epicatechin-(C4-C8)-(+)-catechin) [12]. A-type oligomers arise from B-type equivalents either enzymatically (e.g., through the action of polyphenol oxidase) or chemically (e.g., in the presence of DPPH). In the latter case, a quinone methide mechanism has been evidenced [13]. The once-oxidized cinnamtannins can undergo further oxidation, giving rise to the twice-oxidized trimer ((−)-epicatechin-(C4-C8,C2-O-C7)-(−)-epicatechin-(C4-C8,C2-O-C7)-(−)-epicatechin from B1 and (−)-epicatechin-(C4-C8,C2-O-C7)-(−)-epicatechin-(C4-C8,C2-O-C7)-(+)-catechin from D1).

C1 is partially degraded through chocolate processing, especially during fermentation [14], roasting [15], and conching [16]. Yet when polyphenols are thermally degraded, most derived products are potentially antioxidant. Furthermore, C1 is regenerated from higher procyanidins when the medium is sufficiently liquid for depolymerization (e.g., during liquid conching at 90 °C applied after dry conching at 60 °C [17]). These conditions were selected in a patent aiming at higher antioxidant production [16].

The aim of the present work was to assess how procyanidin C1 degradation products might contribute to the antioxidant activity of a chocolate. First, HPLC-HRMS/MS-ESI(−) was used to evidence the degradation compounds issued from C1 in an aqueous model medium heated at 90 °C. A similar investigation focusing on procyanidin B2 recently showed that in this simple medium, one can readily identify molecules that would be generated in more complex lipidic media [18]. The C1 medium was further analyzed by RP-HPLC-online TEAC to assess the relative antioxidant capacity of each new compound. Finally, mass spectrometry in the SRM mode was used to see if the new compounds occur in raw cocoa beans and to determine their fate during fermentation and roasting.

## 2. Materials and Methods

### 2.1. Chemicals 

Acetonitrile (99.9%) and methanol (99.9%) were supplied by VWR (Leuven, Belgium). Formic acid (99%) was obtained from Acros Organic (Geel, Belgium). (−)-Epicatechin (98%), (+)-catechin (98%), ABTS (2,2′-azino-bis(3-ethylbenzothiazoline-6-sulfonic acid), and Trolox (6-hydroxy-2,5,7,8-tetramethylchromane-2-carboxylic acid) were supplied by Sigma-Aldrich (Bornem, Belgium). (−)-Epicatechin-4-8-(−)-epicatechin (procyanidin B2, 99%) and (−)-epicatechin-4-8-(−)-epicatechin-4-8-(−)-epicatechin (procyanidin C1, 99%) were supplied by PhytoLab GmbH&Co. KG (Vestenbergsgreuth, Germany). Aqueous solutions were made with Milli-Q water (resistance=18 mΩ) (Millipore, Bedford, MA, USA).

### 2.2. Cocoa Samples 

Raw and fermented UF 654 (Trinitario) cocoa beans from Cuba were provided by the Instituto de Investigaciones para la Industria Alimenticia (Habana, Cuba). German Cocoa (Forastero) and ICS 40 (Trinitario) cocoa samples were collected in the area of Ngoumou and Nkomvoene (Cameroon). Forastero beans from Bahia, Brazil, were provided by Le Cercle du Cacao (Brussels, Belgium). The beans were roasted in a ventilated heat chamber (30 min at 150 °C) and allowed to cool down at room temperature before analysis.

### 2.3. Preparation of Procyanidin Model Media 

Aqueous model media were prepared by diluting procyanidin C1 to 1000 mg/L in ultrapure water (10,000 mg/L stock solution prepared in methanol) before heat treatment at 90 °C for 12 h (chosen on the basis of our previous work [18]). After filtration (0.22 µm), the samples were kept at –80 °C before injection.

### 2.4. Extraction of Flavan-3-ols from Cocoa Beans/Chocolate 

This method was adapted from that developed in our laboratory for the analysis of flavan-3-ols in chocolate [19]. All extraction steps were done in duplicate. Seven grams of cocoa beans/chocolate were defatted with diethyl ether (3 × 50 mL) at room temperature under gentle stirring. After centrifugation, the samples were dried under vacuum. Defatted samples spiked with 500 μL kaempferol at 10,000 mg/L in methanol (used as internal standard; 714 mg/kg bean or chocolate weight) were extracted with 3 × 50 mL acetone:water:acetic acid (70:28:2, v/v/v). The three extracts from each sample were kept under nitrogen and then mixed together. The resulting sample was concentrated by rotary evaporation and freeze-dried.

### 2.5. HRMS/MS Identification. 

High-resolution MS/MS analyses were performed on a C18 Prevail column (150 × 2.1 mm, 2.7 µm) (Grace, Deerfield, IL, USA) eluted with a multilinear gradient made with A (water containing 1% acetonitrile and 2% formic acid) and B (acetonitrile containing 2% formic acid). Gradient elution was as follows: from 97% A to 91% in 5 min, 91% to 85% in 25 min, from 85% to 64% in 35 min, from 64% to 10% in 10 min, and isocratic for 20 min at 200 µL/min flow rate. Five microliters of the sample were injected onto the column kept at 20 °C. The Exactive system was composed of Accela LC coupled to the Orbitrap mass spectrometer and controlled with the Xcalibur software version 2.0.7 (Thermo Fisher Scientific, Austin, TX, USA).

### 2.6. RP-HPLC-DAD-ESI(−)-MS/MS Semi Quantitation. 

Quantitations were done by connecting the same column with the same elution program to a SpectraSystem equipped with an AS3000 autosampler and a P4000 quaternary pump. The system was controlled with the Xcalibur software version 1.2 (ThermoFisher). Compounds were monitored from 200 to 800 nm with a UV6000LP diode array detector. Mass spectra were acquired with an LCQ ion trap mass spectrometer equipped with an ESI source (ThermoFisher). Collision-induced dissociation spectra were recorded at a relative collision energy of 30, 35, and 40%, respectively, for singly charged [M−H]^−1^ ions of monomers (*m*/*z* 289), dimers (*m*/*z* 577, 575), and trimers (*m*/*z* 865, 863, 861). The ESI inlet conditions were as follows: source voltage, 4.9 kV; capillary voltage, –4 V; capillary temperature, 200 °C; sheath gas, 39 psi. Semi-quantitations were done with the calibration curves of C1 for all C1-derived compounds except catechin, epicatechin, and B2, for which their own respective calibration curves were used.

### 2.7. RP-HPLC-online TEAC 

RP-HPLC-online TEAC was performed according to the method of Leitao et al. [20] with slight modifications [21]. Contrary to global TEAC, this online method allows the antioxidant capacity of each compound to be evaluated separately after elution on a HPLC column. Ten mg ABTS was dissolved in 2.6 mL of an aqueous solution of potassium persulfate (2.5 mM) (final concentration: 7 mM ABTS^+•^). After 12–16 h storage in the dark at room temperature to ensure the stabilization of the radical, the ABTS^+•^ stock solution was diluted 200 times with ethanol to reach an absorbance of 0.77 ± 0.02 at 642 nm (working solution). The HPLC system (Waters, Milford, MA, USA) consisted of a 1525 binary pump and a 2489 UV/vis detector set at 280 nm. Separation of sample constituents (5 μL) was carried out on the same C18 Prevail column as described above with the same gradient conditions. Data were analyzed with the Breeze 2 software (Waters). After detection at 280 nm, the ABTS^+•^ working solution was added to a reaction coil (stainless steel, 8 m × 0.25 mm) with a mixing tee (flow rate: 100 μL/min). The decolorization occurring when ABTS^+•^ was trapped by a compound in the eluate was monitored with a second 2487 UV/vis detector (Waters) set at 642 nm. The antioxidant activity of each compound was calculated in Trolox equivalents (TE).

## 3. Results and Discussion

### 3.1. Degradation of Procyanidin C1 at 90 °C

**Figure 1:**
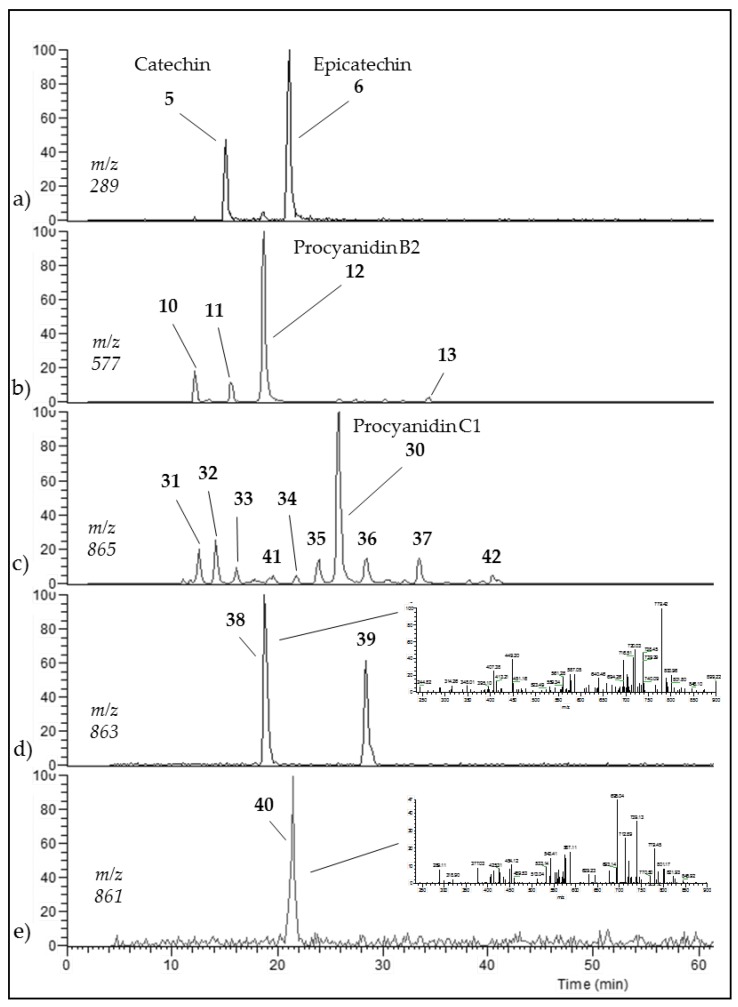
RP-HPLC-MS/MS(ESI(−)) chromatograms, recorded at (**a**) *m*/*z* = 289, (**b**) *m*/*z* = 577, (**c**) *m*/*z* = 865, (**d**) *m*/*z* = 863, and (**e**) *m*/*z* = 861, of procyanidin C1 model medium heated for 12 h at 90 °C. Corresponding structures in Table 1.

An aqueous model medium containing 1000 mg/kg of commercially available procyanidin C1 was subjected to thermal treatment (12 h at 90 °C). As previously observed for B2 [18], depolymerization occurred, leading here to procyanidin B2 (compound **12**) and (−)-epicatechin (**6**). Once created, epimerization also took place both on monomers and on dimers, yielding (−)-catechin (**5**) and epimers of B2 (**10**, **11,** and **13**), respectively (Figure 1a,b).

**Figure 2:**
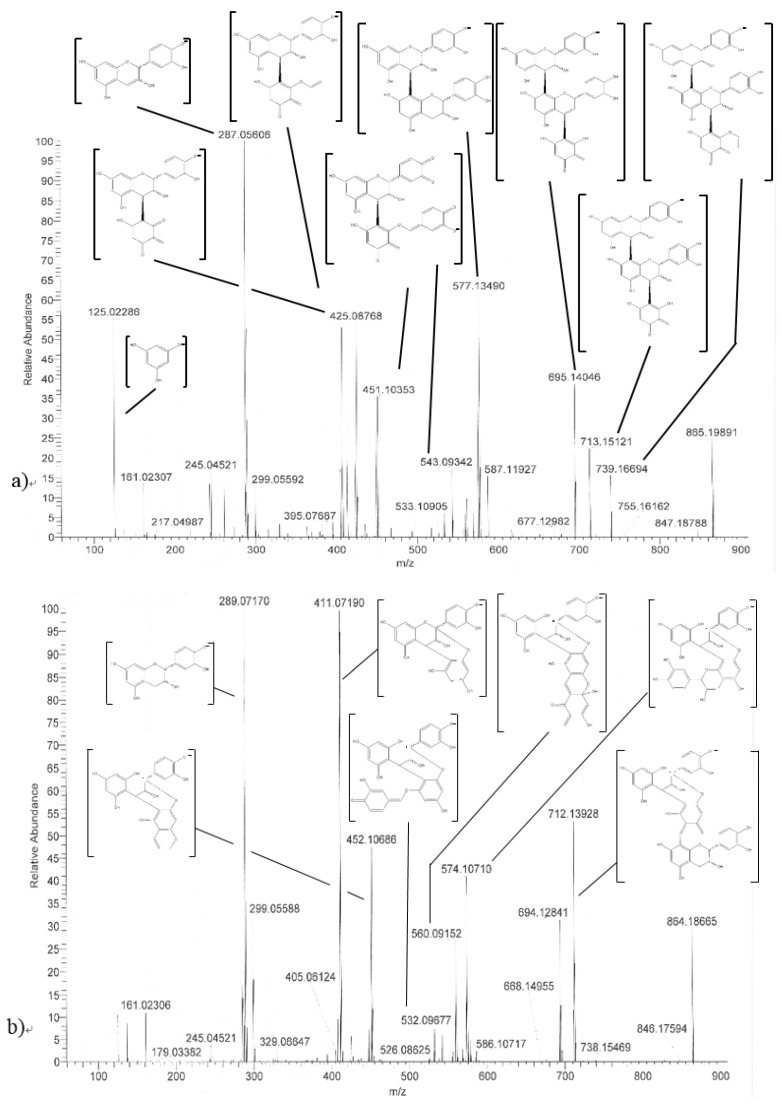
HRMS/MS spectra of (**a**) procyanidin C1 and (**b**) compound 41, with hypothetical structures and fragmentation.

Trimer C1 (**30**) was also found to epimerize, yielding compounds **31** to **37** (Figure 1c), all exhibiting the same HRMS/MS spectrum at *m*/*z* 865 (Figure 2a). As expected, seven peaks were found, accounting for 62% of the initial amount of C1 and corresponding to 3 (−)-epicatechin moieties able to epimerize to (−)-catechin. As these epimers are all commercially unavailable, complete identification remains delicate, even though (−)-catechin epimers probably elute first [17].

Worth stressing is the presence of two other compounds (**41** and **42** in Figure 1c and Table 1) displaying an HRMS/MS spectrum very different from that of C1 and its epimers (Figure 2b). Intense peaks occurred for both compounds at *m*/*z* 411 (411.07190; C_21_H_15_O_9_; δ = 1.82 ppm), 712 (712.13928; C_44_H_24_O_10_; δ = 3.79 ppm), 574 (574.10710; C_37_H_12_0_7_; δ = 4.20 ppm) and 289 (289.07170; C_15_H_13_O_6_; δ = 3.64 ppm), instead of 577 and 287 in C1, indicating that the upper and lower methide quinone fragmentations did not occur in the same way. Hypothetical structures meeting the exact mass requirements are proposed in Table 1 and Figure 2.

As for A2, which has been found in a model B2 degradation medium [18], oxidation occurred on procyanidin C1, leading to two compounds (**38** and **39**, Table 1) monitored in ESI-(−) at *m*/*z* = 863 (Figure 1d). This oxidation occurred either between two extension units ((−)-epicatechin-(C4-C8, C2-O-C7)-(−)-epicatechin-(C4-C8)-(−)-epicatechin, also known as cinnamtannin B1) or with the terminal unit ((−)-epicatechin-(C4-C8)-(-)-epicatechin-(C4-C8, C2-O-C7)-(−)-epicatechin).

If oxidation continues on both once-oxidized trimers, a trimer with *m*/*z* = 861 is formed (40; RT=21.5 min), corresponding to (−)-epicatechin-(C4-C8, C2-O-C7)-(−)-epicatechin-(C4-C8, C2-O-C7)-(−)-epicatechin. (Figure 1e, Table 1).

In model media containing the monomer (-)-epicatechin or the dimer B2, oxidation also led to chemical oligomers (dimers and a trimer), possessing a C2’/5’/6’-C8 interflavan linkage instead of the C4-C8 linkage encountered in native flavan-3-ol oligomers [18]. In the C1 degradation medium, no such oxidation products were found, suggesting that depolymerization/epimerization occurs much more than chemical polymerization.

After 12 h at 90 °C, only 209 mg/kg of procyanidin C1 remained (21%). All *m*/*z* = 865 isomers accounted for up to 47% of the initial C1 concentration, while once-oxidized dimers and twice-oxidized dimers accounted for 7% and 2%, respectively. C1 proved to be slightly more resistant than (−)-epicatechin and dimer B2 [18], (−79% versus −80% and −84%, respectively).

### 3.2. Antioxidant Capacity of C1 Degradation Products

**Figure 3:**
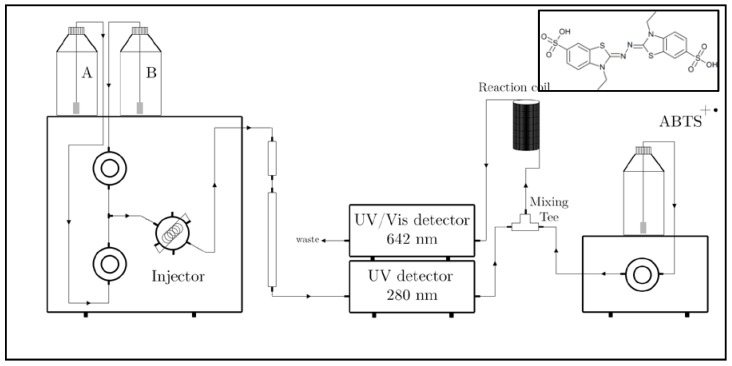
Diagram describing the apparatus used for RP-HPLC-online TEAC.

To assess the antioxidant activity of procyanidin C1 degradation products, the model medium was further analyzed by RP-HPLC hyphenated to online TEAC (Figure 3).

**Figure 4:**
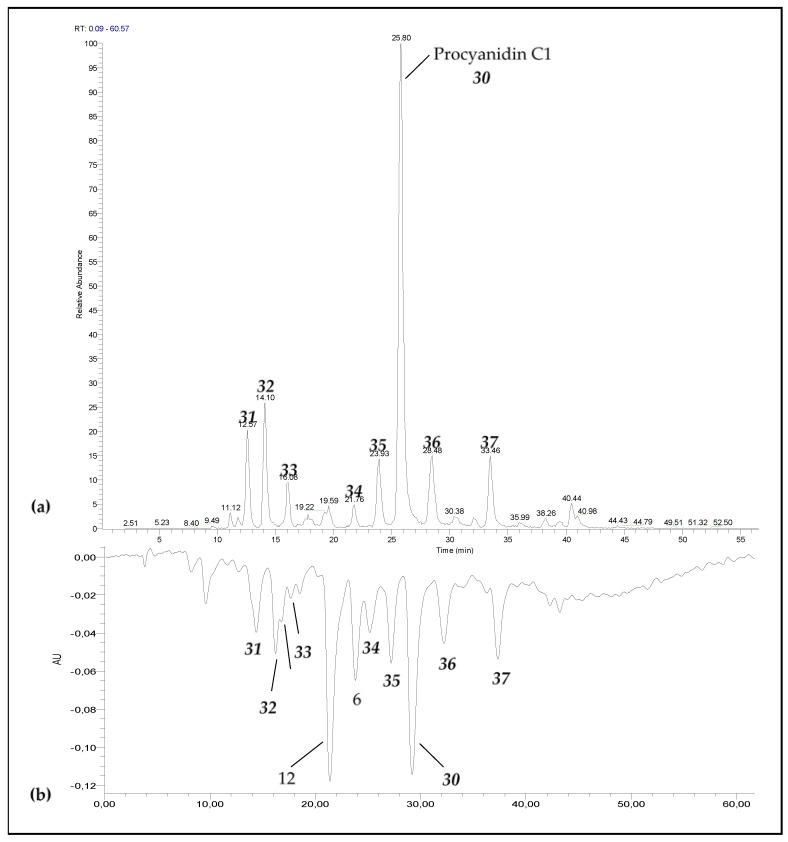
(**a**) RP-HPLC-MSMS(ESI(−)) chromatograms recorded at *m*/*z* = 865, 577, and 289. (**b**) RP-HPLC-online TEAC chromatogram after the reaction with ABTS^+•^ (642 nm) for procyanidin C1 model medium heated for 12 h at 90 °C.

As shown in Figure 4, catechin, epicatechin, B2, and all the peaks detected at *m*/*z* 865 exhibit antioxidant activity. As shown in Table 2, the antioxidant activity of most degradation products was very close to that of C1. As epimerization affects neither the catechol moiety nor the C5/C7 OH groups on the A-ring (both recognized as sites of antioxidant capacity), similar TEAC values were found. (−)-Catechin (**5**), (−)-epicatechin (**6**), and procyanidin B2 (**12**)) exhibited lower TEAC values when expressed in μM TE/μM but similar values when expressed in μM TE/mg·kg^–1^.

### 3.3. Occurrence of C1 Degradation Compounds in Cocoa Beans and Chocolate

**Figure 5:**
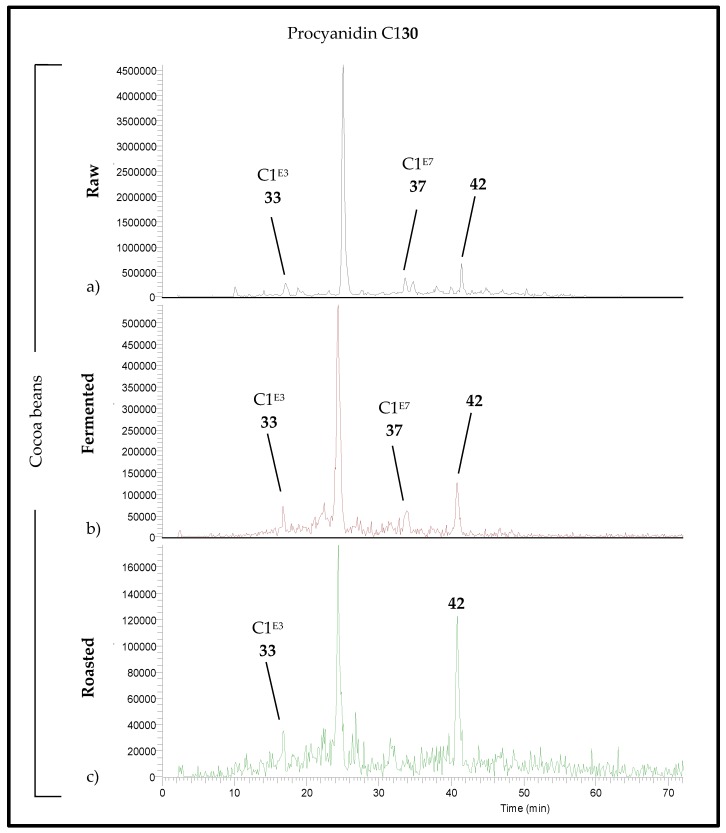
RP-HPLC-MS/MS(ESI(−)) chromatograms of extracts of (**a**) raw (*m*/*z* = 865), (**b**) fermented (*m*/*z* = 865), and (**c**) roasted (*m*/*z* = 861, 863, and 865) Criollo cocoa beans from Cuba (C 411).

**Figure 6:**
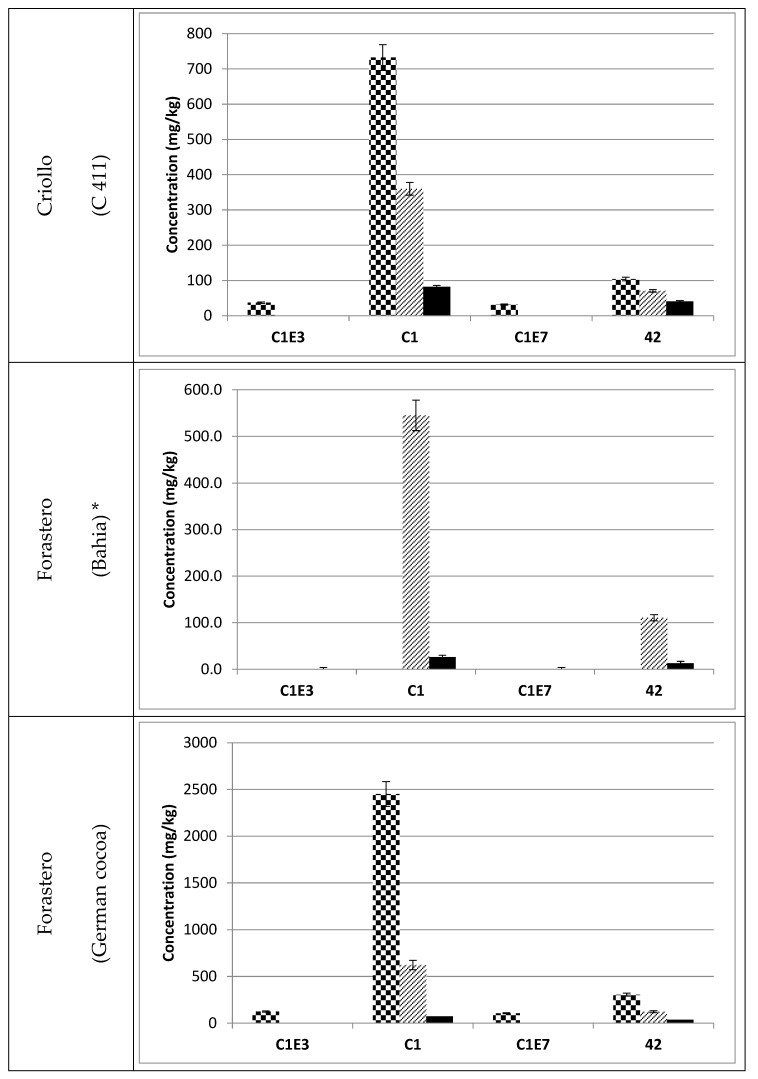

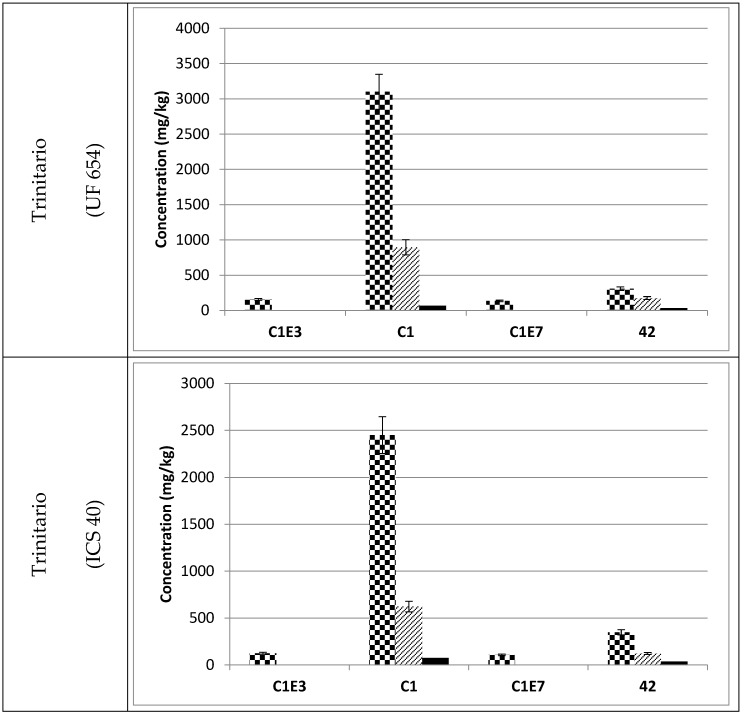
Concentrations of procyanidin C1 and its isomers in raw 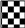, fermented 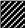 and roasted (30 min—150 °C) 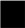 cocoa beans. * Raw beans unavailable.

In all the raw cocoa beans investigated here, RP-HPLC analyses showed that in addition to procyanidin C1 (30), two epimers (C1E3 (33) and C1E7 (37)) and one isomer (42) were found (Figure 5a). The concentration of the epimers and isomer dropped strongly during fermentation (Figure 5b and Figure 6) and subsequent roasting (Figure 5c and Figure 6): C1E7 (37) completely disappeared while C1E3 (33) was no longer quantifiable. Compound 42 was less degraded.

**Figure 7:**
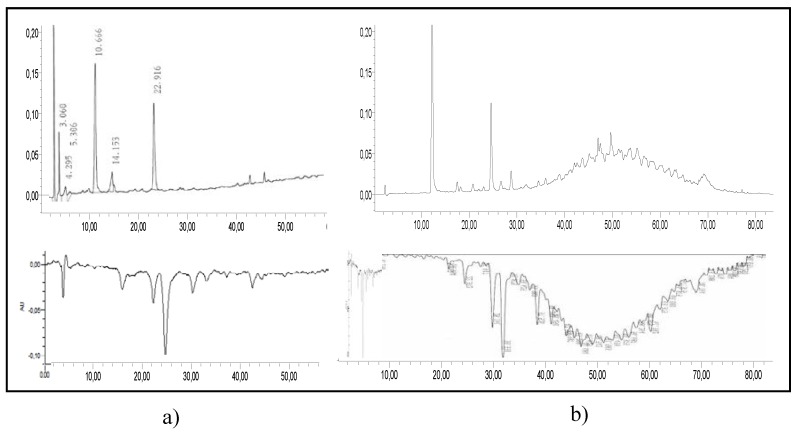
RP-HPLC-online TEAC chromatogram before (280 nm, above) and after (642 nm, below) the reaction with ABTS^+•^ of (**a**) fermented dried beans and (**b**) chocolate extract.

In fermented dried beans (Figure 7a), C1 and its smaller degradation products here evidenced exhibited antioxidant activity. In chocolate (Figure 7b), the loss of flavan-3-ols is obvious in regard of the methylxanthines (caffeine and theobromine), but the same compounds as recorded before still showed antioxidant activity. Furthermore, an interesting pool of antioxidant compounds appeared and issued from the chocolate manufacturing. This pool eluted much later on our C18 column, indicating the relative lower polarity of its constituents. Therefore, we suspect these molecules to be oxidation products of native cocoa oligomers. As it was shown with our C1 model medium, oxidation does not lead to polymerized compounds with more than three units linked chemically. However, intra-oxidation is a much favored way leading to new interflavane linkages within native bigger oligomers present in cocoa and hence absent from our model medium.

## 4. Conclusions

In conclusion, we observed here that epimerization is the main reaction involved in the degradation of procyanidin C1 (62% of degradation products). Depolymerization is the second main degradation pathway while oxidation affected it to a lesser extent. This last mechanism led to once- and twice-oxidized dimers while no chemical oligomer involving the native trimer was found, neither in the model medium nor in the cocoa. Yet, two C1 isomers were retrieved in the beans. Finally, RP-HPLC-Online TEAC allowed us to evidence that C1 degradation products showed an antioxidant activity close to that of C1 (when expressed in µM TE/mg·kg^−1^).

## Figures and Tables

**Table 1:** Structure and MS/MS-ESI(−) fragmentation of degradation products formed in procyanidin C1 model medium heated for 12 h at 90 °C.

Name (peak)	Retention Time (min)	(M−H)^−1^	Structure	MS/MS
(−)-Catechin (5)	15.0	289	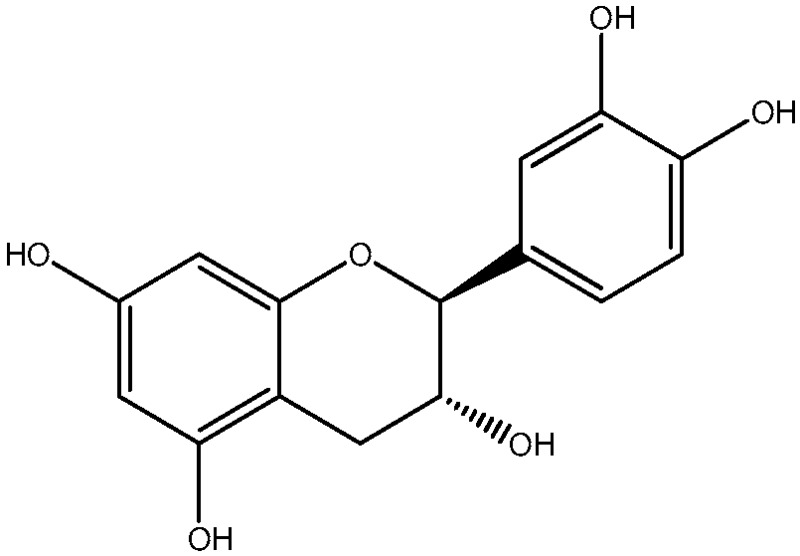	289.1 (100) 245.1 (20) 179.1 (6)
(−)-Epicatechin (6)	21.1	289	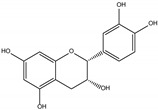	289.1 (100) 245.1 (20) 179.1 (6)
Procyanidin B2 epimer (10,11,13)	12.2 15.2 25.7	577	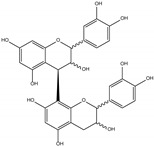	425.1 (100) 451.1 (65) 407.2 (47) 289.1 (28)
Procyanidin B2 (12)	18.7	577	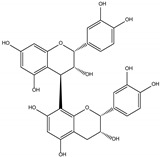	425.1 (100) 451.1 (65) 407.2 (47) 289.1 (28)
Procyanidin C1 (30)	25.8	865	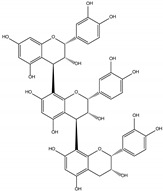	695.1 (100) 577.1 (64) 739.1 (49) 713.1 (36) 407.2 (29) 575 (25) 425 (21)
Procyanidin C1 epimer (31–37)	12.6 14.1 15.8 21.8 23.9 28.5 33.5	865	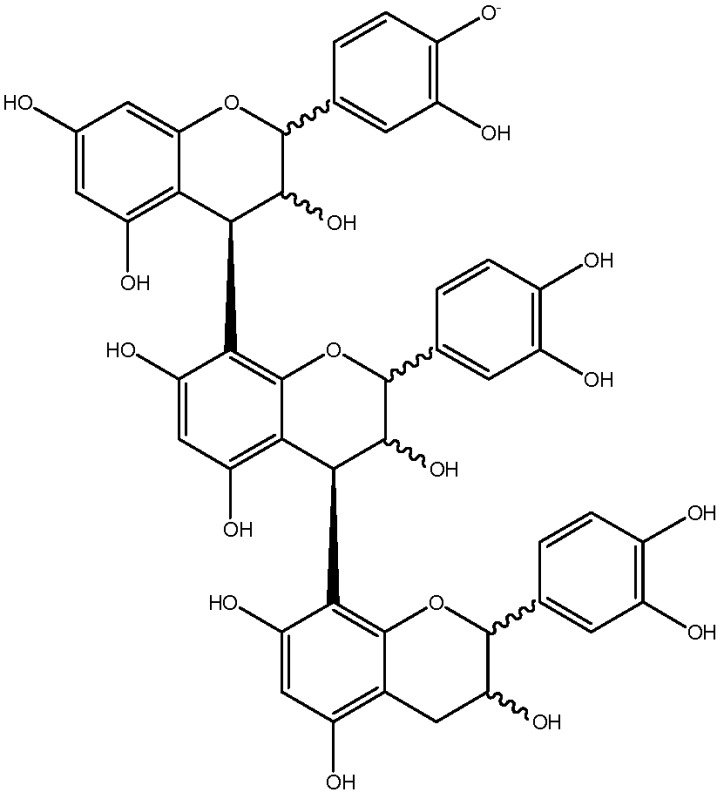	695.1 (100) 577.1 (64) 739.1 (49) 713.1 (36) 407.2 (29) 575 (25) 425 (21)
Once-oxidized trimer (38,39)	18.7 28.4	863	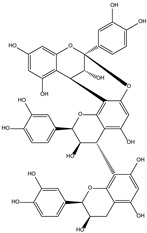 or 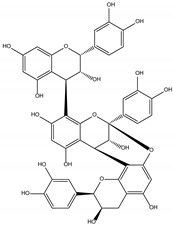	845.1 (100) 575.1 (78) 289.2 (63) 287.1 (59) 125.1 (28)
Twice-oxidized trimer (40)	21.5	861	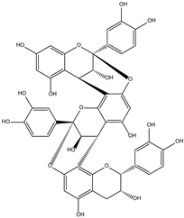	843.1 (100), 691.2 (79), 649.1 (38), 575.1 (21).
C1 isomer (41,42)	20.8 40.4	865	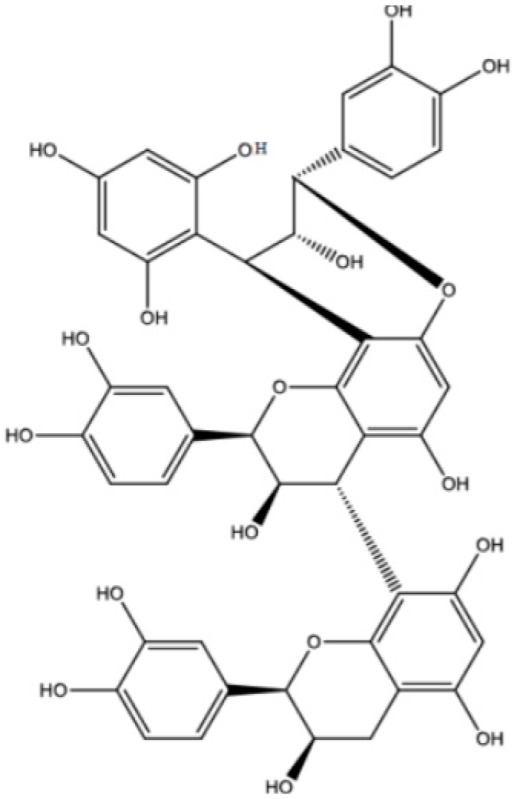	289.1 (100), 411.1 (98), 721.1 (53), 574.1 (41), 560.1 (31).

**Table 2:** Antioxidant capacity of degradation products formed in procyanidin C1 model medium heated for 12 h at 90 °C.

Compound (peak)	Source	TEAC	
		µM TE/µM	µM TE/mg·kg^−1^
Catechin (5)	a	0.6	2.3
Epicatehin (6)	a	0.7	2.5
Procyanidin B2 (12)	a	1.3	2.4
Procyanidin C1 (30)	a.b	2.2	2.5
C1^E1^ (31)	b	2.1	2.5
C1^E2^ (32)	b	2.2	2.6
C1^E3^ (33)	b	*	*
C1^E4^ (34)	b	2.1	2.5
C1^E5^ (35)	b	2.3	2.6
C1^E6^ (36)	b	2.0	2.4
C1^E7^ (37)	b	2.2	2.5
38 and 39	b	*	*
40	b	*	*
41 and 42	b	*	*
Trolox	a	1	4

**a** = injection of commercial standard; **b** = issued from model medium; * unresolved in online TEAC.

## Abbreviations

RP-HPLC-HRMS/MS: reversed-phase high-performance liquid chromatographic
HRMS/MS: high-resolution mass pectrometry/mass pectrometry
ESI: electrospray ionization
TEAC: Trolox equivalent antioxidant capacity
DPPH: diphenylpicrylhydrazyl
SRM: selected reaction-monitoring
